# Supplementary material for: Trait anxiety is associated with hidden state inference during aversive reversal learning
Source: Nat Commun. 2023 Jul 14;14:4203. doi: 10.1038/s41467-023-39825-3 (PMC10349120; doi:10.1038/s41467-023-39825-3)
Supplement: Supplementary file 3 — Reporting Summary [file 41467_2023_39825_MOESM3_ESM.pdf]

nature portfolio

Corresponding author(s): Schuck, W. Nicolas; Zika, Ondrej

Last updated by author(s): 30/03/2023

Reporting Summary

Nature Portfolio wishes to improve the reproducibility of the work that we publish. This form provides structure for consistency and transparency in reporting. For further information on Nature Portfolio policies, see our Editorial Policies and the Editorial Policy Checklist.

Statistics

For all statistical analyses, confirm that the following items are present in the figure legend, table legend, main text, or Methods section.

n/a	Confirmed	
☐	☒	The exact sample size (*n*) for each experimental group/condition, given as a discrete number and unit of measurement
☐	☒	A statement on whether measurements were taken from distinct samples or whether the same sample was measured repeatedly
☐	☒	The statistical test(s) used AND whether they are one- or two-sided<br>*Only common tests should be described solely by name; describe more complex techniques in the Methods section.*
☐	☒	A description of all covariates tested
☐	☒	A description of any assumptions or corrections, such as tests of normality and adjustment for multiple comparisons
☐	☒	A full description of the statistical parameters including central tendency (e.g. means) or other basic estimates (e.g. regression coefficient) AND variation (e.g. standard deviation) or associated estimates of uncertainty (e.g. confidence intervals)
☐	☒	For null hypothesis testing, the test statistic (e.g. *F*, *t*, *r*) with confidence intervals, effect sizes, degrees of freedom and *P* value noted<br>*Give P values as exact values whenever suitable.*
☒	☐	For Bayesian analysis, information on the choice of priors and Markov chain Monte Carlo settings
☒	☐	For hierarchical and complex designs, identification of the appropriate level for tests and full reporting of outcomes
☐	☒	Estimates of effect sizes (e.g. Cohen's *d*, Pearson's *r*), indicating how they were calculated

Our web collection on statistics for biologists contains articles on many of the points above.

Software and code

Policy information about availability of computer code

Data collection	Matlab 2016a; Psychtoolbox 3, LimeSurvey
Data analysis	Matlab 2019a; R 3.6.3, Full list of packages and their versions is available in the associated repository in the renv.lock file: https://github.com/ozika/trait-anxiety-and-state-inference-zika2023. For model fitting we used Bayesian Adaptive Direct Search (BADS) algorithm.

For manuscripts utilizing custom algorithms or software that are central to the research but not yet described in published literature, software must be made available to editors and reviewers. We strongly encourage code deposition in a community repository (e.g. GitHub). See the Nature Portfolio guidelines for submitting code & software for further information.

Data

Policy information about availability of data

All manuscripts must include a data availability statement. This statement should provide the following information, where applicable:

- Accession codes, unique identifiers, or web links for publicly available datasets
- A description of any restrictions on data availability
- For clinical datasets or third party data, please ensure that the statement adheres to our policy

All data and code associated with the analyses in this paper are available on-line: https://github.com/ozika/trait-anxiety-and-state-inference-zika2023
Full data and code availability statements are in the main manuscript.

Human research participants

Policy information about studies involving human research participants and Sex and Gender in Research.

Reporting on sex and gender	Participants were asked to self-report their gender. This variable was used to characterize the population but not as a variable-of-interest or a covariate in the analyses.
Population characteristics	Out of the total 89 participants, 47 were Female and 42 Male. The mean age was 25.5 years.
Recruitment	Participants were recruited using local advertisement (leaflets) and the SONA recruitment system (local recruitment database).
Ethics oversight	All three studies were approved by the Central University Research Ethics Committee of Oxford University (R44738/RE001; R29583/RE004; R52892/RE001)

Note that full information on the approval of the study protocol must also be provided in the manuscript.

Field-specific reporting

Please select the one below that is the best fit for your research. If you are not sure, read the appropriate sections before making your selection.

☐ Life sciences ☒ Behavioural & social sciences ☐ Ecological, evolutionary & environmental sciences

For a reference copy of the document with all sections, see nature.com/documents/nr-reporting-summary-flat.pdf

Behavioural & social sciences study design

All studies must disclose on these points even when the disclosure is negative.

Study description	Quantitative experimental design. Main data consisted of participants shock expectancy ratings.
Research sample	Oxford residents recruited via advertisement (mostly general public) and sona system (mostly students). 89 individuals took part in the three studies (47 female, mean age 25.5). The sample was not assessed for representativeness. The goal was to recruit random sample from general public composed of individuals without current psychiatric diagnosis and off psychoactive medication.
Sampling strategy	The data set consists of three separate studies for which separate power calculations were performed. The data sets were pooled to further increase statistical power. Random sampling strategy was used.
Data collection	Data were collected in laboratory settings. Study 1 involved scanning in the MRI scanner. Study 2 involved measurement of pupil dilation. Questionnaires were collected either by pen and paper (Study 2) or using web interface (Studies 1 and 3). Task was administered using a Windows 7 PC. Electrical stimuli were delivered using Digitimer D7A stimulator, individual intensity was calibrated to 8/10 level. Experimenter was present in the testing room only during preparatory procedures, during the performance of the task the participant was alone. In Study 2, the design was double-blinded, so the researcher was not aware of group assignment. Researchers conducting the studies were only marginally aware of the research question, but no active steps were taken to blind them.
Timing	Study 1: 05/2016 - 02/2017<br>Study 2: 03/2017 - 03/2018<br>Study 3: 12/2017 - 04/2018
Data exclusions	In Study 2 (double-blind study), half of the participants received Angiotensin-II inhibitor losartan, those were excluded from the sample. Full data of four participants and partial data of two participants were excluded due to missing behavioural data (presentation computer or stimulator stopped working during the task). Two participants were excluded because of missing anxiety scores. The total number of participants included was 89.
Non-participation	One participant was worried about the electrical stimulation and decided to withdraw prior to the initiation of the session.
Randomization	Experimental schedules and cues were assigned randomly to participants.

Reporting for specific materials, systems and methods

March 2021

We require information from authors about some types of materials, experimental systems and methods used in many studies. Here, indicate whether each material, system or method listed is relevant to your study. If you are not sure if a list item applies to your research, read the appropriate section before selecting a response.

Materials & experimental systems

n/a	Involved in the study
☒	☐ Antibodies
☒	☐ Eukaryotic cell lines
☒	☐ Palaeontology and archaeology
☒	☐ Animals and other organisms
☒	☐ Clinical data
☒	☐ Dual use research of concern

Methods

n/a	Involved in the study
☒	☐ ChIP-seq
☒	☐ Flow cytometry
☒	☐ MRI-based neuroimaging
